# MFDSU-Net: a novel semantic-geometric collaborative network for cattle segmentation in complex farm environments

**DOI:** 10.3389/fvets.2026.1866409

**Published:** 2026-06-10

**Authors:** Wangli Hao, Yufan Jiang, Yifei Liu, Jingrong Wang, Meng Han, Yanhong Liu, Fuzhong Li

**Affiliations:** 1 Faculty of Software Technologies, Shanxi Agricultural University, Jinzhong, Shanxi, China; 2 School of Information Science and Engineering, Shanxi Agricultural University, Jinzhong, Shanxi, China

**Keywords:** cattle segmentation, MDSBlock, MFABlock, precision livestock farming, semantic-geometric, U-Net

## Abstract

Cattle segmentation in precision livestock farming faces persistent challenges from background-induced semantic uncertainty and occlusion-driven geometric distortion. To address these issues, this paper proposes MFDSU-Net, a novel U-Net architecture that jointly refines semantic and geometric representations. The MFDSU-Net architecture comprises two core modules, including the Multi-Scale Feature Aggregation Block (MFABlock) and the Multi-scale Deformation Sampling Block (MDSBlock) respectively. Specifically, MFABlock captures contextual information across varying receptive fields, enhancing model robustness against complex backgrounds and scale variations. Concurrently, MDSBlock adaptively models spatial deformations, preserving boundary fidelity against occlusions and pose variations. Experimental results show that the proposed MFDSU-Net achieves a Dice of 88.14% and an IoU of 81.38%, outperforming existing state-of-the-art models by approximately 0.5% and 0.8%. Furthermore, with only 0.7M parameters and 2.8 GFLOPs, MFDSU-Net maintains high inference efficiency, rendering it suitable for real-time deployment on resource-limited agricultural edge devices.

## 1 Introduction

Cattle segmentation serves as a core component of visual perception in intelligent livestock farming systems. Its accuracy directly determines the reliability of downstream tasks, including cattle counting, behavior analysis, and health assessment (1–3). As the global livestock industry undergoes rapid tech-driven evolution, there is an increasing demand for lightweight, real-time AI solutions that can be deployed on edge devices in complex farm environments (4). Consequently, achieving high precision segmentation is of paramount importance for enhancing the automation level and production efficiency of pasture management (5). Nevertheless, cattle segmentation in real-world farm environments faces two fundamental challenges. First, complex and variable background textures, coupled with inherent variations in target scale and pose, frequently lead to insufficient feature extraction. This induces semantic confusion, hindering the model from accurately distinguishing cattle foreground from cluttered backgrounds (6). Second, frequent mutual occlusion among cattle and inherent blurring of target boundaries severely disrupt the accurate delineation of object contours, resulting in geometric distortion that causes segmentation boundaries to deviate from the true target morphology (7–9).

Beyond cattle-focused segmentation studies, several studies on pig visual perception and infrared-visible image fusion provide useful methodological references for livestock image analysis. For instance, the INPC model achieves accurate instance segmentation of pigs in infrared images, demonstrating the feasibility of deep learning-based livestock target extraction under challenging imaging conditions (10). Another study performs non-contact pig body temperature measurement through ear segmentation and multi-factor infrared temperature compensation, highlighting the importance of accurate segmentation for downstream phenotypic measurement (11). However, these studies are mainly designed for pig-related infrared perception and do not explicitly address cattle-specific challenges, such as complex fence backgrounds, inter-object occlusion, and non-rigid geometric deformation.

Infrared and visible image fusion methods, including CLTE, CrossMamba, and LACT-Fusion, have also been developed to improve image representation under complex lighting conditions (12–14). These methods provide useful insights into robust feature enhancement, but their primary focus is cross-modal feature alignment and texture enhancement rather than semantic-geometric collaborative modeling for cattle segmentation. Therefore, further exploration is still needed to develop a lightweight cattle segmentation network that jointly improves semantic discrimination and geometric boundary modeling.

To address the aforementioned challenges, existing research has mainly evolved along three directions: models based on convolutional neural networks (CNNs), which excel at local feature extraction; Transformer-based models that model global dependencies via the self-attention mechanism; and Mamba-based models with linear computational complexity for efficient sequence modeling.

Within the CNN-based technical framework, the U-Net model proposed by Ronneberger et al. (15) pioneered and established the core design paradigm of the symmetric encoder-decoder architecture. This model extracts high-level semantic information through encoder downsampling, and recovers fine-grained spatial details via the upsampling process of the decoder. It maintains outstanding performance even in scenarios with limited annotated data, making it a classic benchmark for cattle segmentation tasks with both high accuracy and practicality. Aiming at the inherent limitations of U-Net, subsequent studies have carried out a series of optimization works. Oktay et al. (16) introduced an attention gating mechanism and proposed Attention U-Net to strengthen the model's ability to focus on cattle target regions. Zhou et al. (17) constructed U-Net++ through a nested decoder architecture to enhance the effect of multi-scale feature fusion. Dinh et al. (18) proposed U-Lite, which takes depth-wise separable convolution as the core design and indicatively develops an axial depth-wise convolution module, providing an important guideline for the design of novel CNN segmentation models. These improvements have achieved performance gains in multiple segmentation tasks, further verifying the core advantages and expansion potential of the U-Net architecture in the field of cattle segmentation.

In contrast, the Transformer architecture has garnered widespread attention for its powerful global context modeling capability, with numerous breakthroughs achieved in the computer vision domain. The foundational Transformer proposed by Vaswani et al. (19) introduced the core self-attention mechanism; subsequently, Dosovitskiy et al. (20) developed the Vision Transformer (ViT), the first to comprehensively apply this mechanism to image tasks, enabling effective capture of global pixel-wise correlations. Furthermore, Chen et al. (21) (TransU-Net) and Liu et al. (22) (SwinU-Net) integrated Transformer blocks into the U-Net architecture, improving the global structure perception of complex targets in segmentation tasks to a certain extent. Despite these advances, the softmax self-attention mechanism in Transformer models has inherent quadratic computational complexity. When processing high-resolution cattle images from real farm scenarios, this mechanism inevitably leads to a substantial increase in memory footprint and excessive inference latency, making it unsuitable for real-time segmentation deployment on resource-constrained edge devices (23).

To balance modeling capability and inference efficiency, novel architectures with linear computational complexity have become a research hotspot. Mamba, proposed by Gu et al. (24), as a state space model with linear scaling, breaks through the computational bottleneck of traditional attention mechanisms. VisionMamba developed by Zhu et al. (25) successfully adapted it to visual tasks and achieved effective capture of bidirectional features. MambaULite by Nguyen et al. (26) further integrates the advantages of Mamba and CNNs, achieving competitive performance with an extremely small number of parameters. In particular, CBAM is introduced into the skip connections to adaptively recalibrate feature representations (27), where channel and spatial attention are jointly exploited to enhance informative features while suppressing irrelevant responses. However, such methods still exhibit limited capability in distinguishing foreground from background in cattle segmentation tasks under complex farm scenarios, such as blurred boundaries and severe occlusions, resulting in inferior segmentation accuracy and robustness compared to U-Net-based models.

Despite the significant progress achieved by existing methods, they still have limitations when dealing with highly dynamic and complex real-world farm environments (28–30). An ideal cattle segmentation model should simultaneously exhibit robust semantic understanding to mitigate complex background interference and precise geometric modeling to address occlusion and deformation. However, existing methods often treat these two capabilities in isolation: CNNs excel at capturing local geometric details but have limited semantic receptive fields; Transformers are good at global semantic modeling but produce coarse geometric details; and Mamba-based models prioritize inference efficiency at the cost of spatial precision. Notably, existing lightweight models such as MambaU-Lite and U-Lite are primarily developed for general object segmentation tasks that are optimized for rigid targets with fixed geometric features and relatively uniform backgrounds. In contrast, cattle segmentation in intensive farm environments presents three distinct challenges: non-rigid targets exhibiting posture variations, cluttered and dynamically changing backgrounds, and frequent inter-object occlusion. None of these challenges have been adequately addressed by general-purpose models, which lack the adaptive spatial sampling capability necessary to capture the irregular contour deformation of cattle, resulting in boundary blurring and segmentation errors in complex farm scenarios. Therefore, under resource-constrained practical deployment conditions, there is an urgent need for a segmentation model that can collaboratively enhance Semantic-Geometric Collaborative.

To address these challenges, this study proposes MFDSU-Net, a lightweight semantic-geometric collaboratively enhanced U-Net framework for cattle segmentation, and investigates the following research questions:

How can semantic representation enhancement and geometric structure modeling be effectively integrated within a lightweight cattle segmentation framework?

How do multi-scale contextual aggregation and adaptive deformable sampling contribute to segmentation robustness under complex farm environments?

Can a lightweight segmentation network maintain competitive segmentation accuracy while reducing computational complexity for potential deployment in resource-constrained agricultural monitoring systems?

The main contributions of this study are summarized as follows:

1. This paper proposes the MFDSU-Net model for precise cattle segmentation, which jointly optimizes semantic feature extraction and object contour modeling. Specifically, the model enhances semantic discrimination through multi-scale context aggregation combined with global structural modeling. It refines boundary delineation via adaptive spatial sampling designed to accommodate deformation and occlusion.
2. A novel MFABlock module is developed to aggregate multi-scale contextual features for cattle segmentation. This module improves adaptability to diverse cattle body shapes, enabling the model to capture subtle contour differences and mitigating semantic ambiguity arising from complex farm backgrounds.
3. The MDSBlock module is introduced to refine contour features in cattle segmentation. It performs adaptive deformation modeling to dynamically adjust the sampling grid. As a result, the model achieves notable performance improvements and effectively addresses challenges related to posture variations and local occlusion.

The remainder of this paper is organized as follows: Section 2 outlines the methods used in our study. Section 3 presents the Dataset and implementation Details. Section 4 depicts the experimental and analysis in detail. Section 5 provides a detailed discussion of the findings. Finally, conclusions are presented in Section 6.

## 2 Method

### 2.1 MFDSU-Net

**Figure 1:**
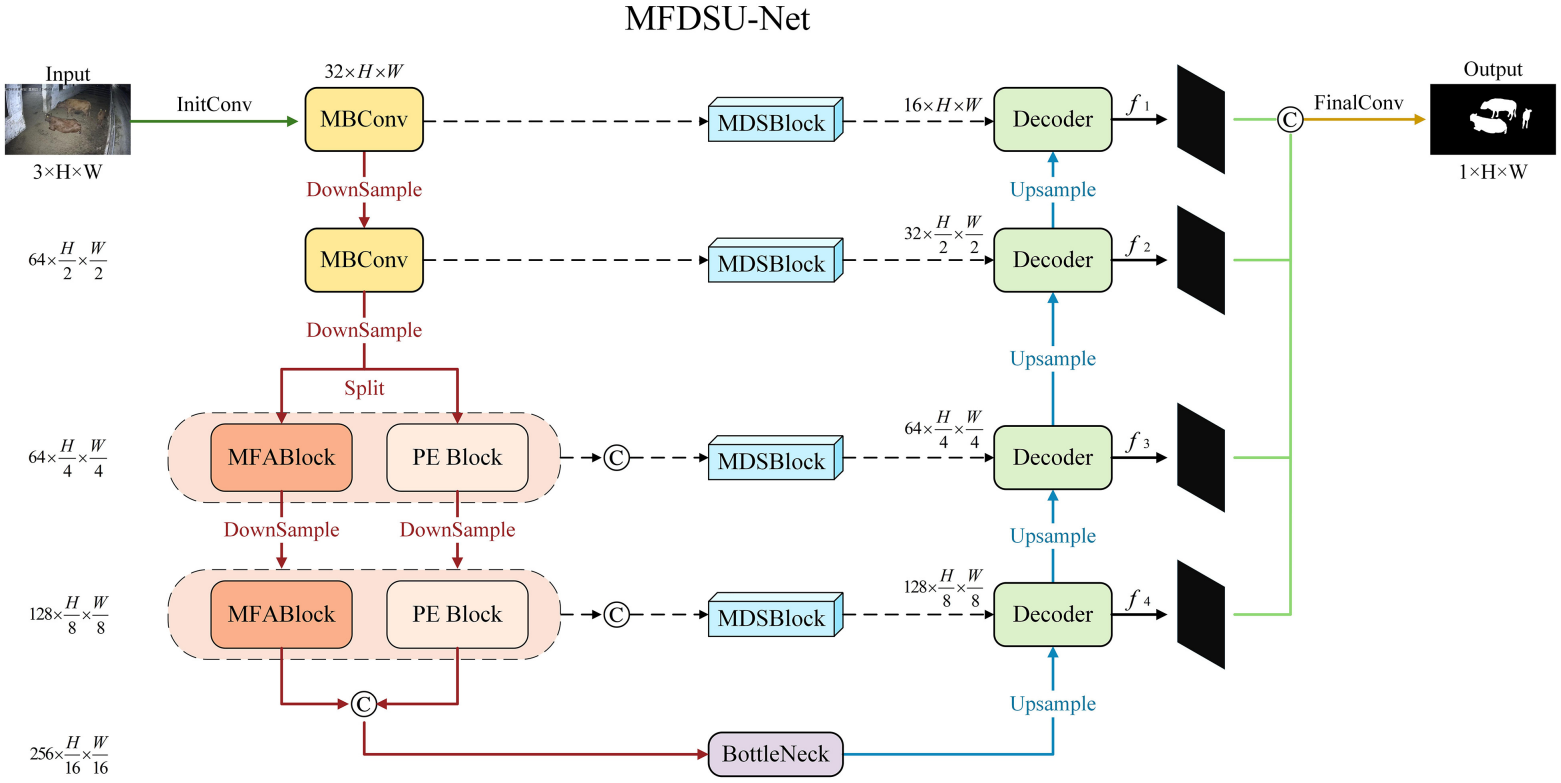
Overall architecture of MFDSU-Net.

MFDSU-Net adopts the classical encoder-decoder structure of U-Net as its backbone. As illustrated in Figure 1, the architecture comprises an input convolution, a four-stage encoder with progressive downsampling, a bottleneck layer, a four-stage decoder with progressive upsampling, and a final output convolution. The encoder stages increase channel dimensions from 16 to 32, 64, and 128, while the decoder stages progressively restore spatial resolution. Skip connections transmit encoder features to corresponding decoder stages to preserve spatial details.

Specifically, the P-Mamba module and the DecoderBlock proposed by Nguyen et al. (26) is incorporated into both the Encoder and Decoder. Meanwhile, the Integrated Channel-Spatial Attention (ICSA) module introduced by Le et al. (31) is adopted in the BottleNeck to enhance feature representation.

Two core modules constitute the primary innovations of this architecture. Specifically, a Multi-scale Feature Aggregation Block is embedded in the third and fourth encoder stages to enhance semantic discrimination. Additionally, a Multi-scale Deformation Sampling Block is inserted in each skip connection to refine boundary delineation. The following subsections detail the design and functionality of these two modules.

### 2.2 Multi-scale feature aggregation block: MFABlock

**Figure 2:**
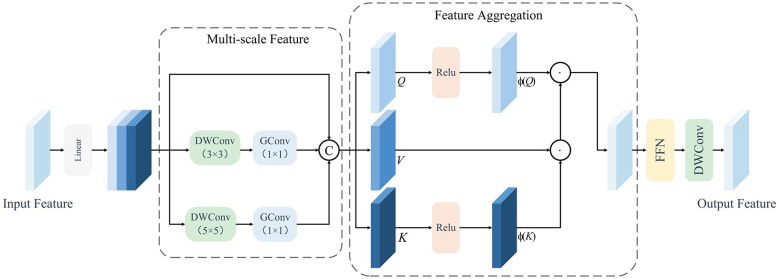
The MFABlock architecture diagram.

Embedded in the third and fourth encoder stages, the Multi-scale Feature Aggregation Block aggregates contextual information across multiple scales to strengthen foreground-background discrimination. As illustrated in Figure 2.

Given the input feature tensor $X\in {\mathbb{R}}^{B\times C_{in}\times H\times W}$, where *B* denotes the batch size, *C*_*in*_ is the number of input channels, and *H* and *W* represent the spatial height and width of the feature map, respectively. The input is first projected to the target channel dimension via a 1 × 1 linear projection layer, yielding the projected feature $X_{proj}\in {\mathbb{R}}^{B\times C\times H\times W}$ which provides a unified feature representation for subsequent multi-scale feature generation and attention computation.

To achieve efficient aggregation of multi-scale contextual information, this module designs a hardware-friendly multi-branch parallel depth-wise convolution structure. It adopts group convolution to fuse multi-scale depth-wise convolution (DWConv) operations, which reduces computational redundancy and improves hardware execution efficiency, while capturing features under different receptive fields. Specifically, parallel DWConv branches with kernel sizes of 3 × 3 and 5 × 5 are designed to capture fine-grained local edge details and coarse-grained global semantic context, respectively. Each DWConv branch is followed by a 1 × 1 group convolution (GConv) for channel-wise feature refinement and interaction:


$$\begin{array}{l} F_{3\times 3}=\text{GCon}{\text{v}}_{1\times 1}\left(\text{DWCon}{\text{v}}_{3\times 3}\left(X_{proj}\right)\right) \end{array}$$ (1)

$$\begin{array}{l} F_{5\times 5}=\text{GCon}{\text{v}}_{1\times 1}\left(\text{DWCon}{\text{v}}_{5\times 5}\left(X_{proj}\right)\right) \end{array}$$ (2)

where DWConv_*k* × *k*_ denotes the depth-wise convolution with kernel size *k* × *k*. The original projected feature and the multi-scale features from the two branches are concatenated along the channel dimension to generate the multi-scale fused feature $F_{multi}\in {\mathbb{R}}^{B\times C\times H\times W}$, realizing complementary fusion of features across different scales and providing multi-scale semantic representation for subsequent global attention modeling.

Aiming at the problem that the computational complexity of traditional softmax self-attention grows quadratically with the feature map resolution, this module designs a ReLU kernel-based linear attention mechanism. By leveraging the associative property of matrix multiplication, it reduces the computational complexity from *O*(*H*^2^*W*^2^) to linear *O*(*HW*), and eliminates hardware-unfriendly operators such as softmax, which greatly improves the inference efficiency of the model when processing high-resolution cattle images on agricultural edge devices. First, the multi-scale fused feature *F*_*multi*_ is projected via 1 × 1 convolutions to generate the query (*Q*), key (*K*), and value (*V*) representations. Then, ReLU is used as the kernel activation function to satisfy the positive definiteness requirement for linear attention decomposition, and the kernel transformation is performed on *Q* and *K*:


$$\begin{array}{l} \phi \left(Q_{s}\right)=\text{ReLU}\left(Q\right),\;\phi \left(K_{s}\right)=\text{ReLU}\left(K\right) \end{array}$$ (3)

where ϕ(·) denotes the kernel transformation function. Linear attention is computed as:


$$\begin{array}{l} O_{s}=\frac{\phi \left(Q_{s}\right)\left(\phi {\left(K_{s}\right)}^{⊤}V\right)}{\phi \left(Q_{s}\right)\left(\phi {\left(K_{s}\right)}^{⊤}\text{1}\right)+\epsilon }, \end{array}$$ (4)

where **1** denotes an all-ones vector with matching dimensions, and ϵ = 10^−15^ is a small constant to avoid numerical instability. This formula only needs to compute $\phi {\left(K_{s}\right)}^{T}V$ and $\phi {\left(K_{s}\right)}^{T}\text{1}$ once, which can be reused across all queries. It completely avoids the high-complexity pairwise similarity calculation between tokens in traditional self-attention, and has significant efficiency advantages in high-resolution cattle segmentation scenarios.

To compensate for the insufficient local feature extraction capability of linear attention, this module introduces a feed-forward network integrated with depth-wise convolution (FFN+DWConv) after the attention output, where the aggregated attention feature is sequentially processed by the FFN and DWConv layers to achieve fine-grained feature optimization.

### 2.3 Multi-scale deformable sampling block: MDSBlock

**Figure 3:**
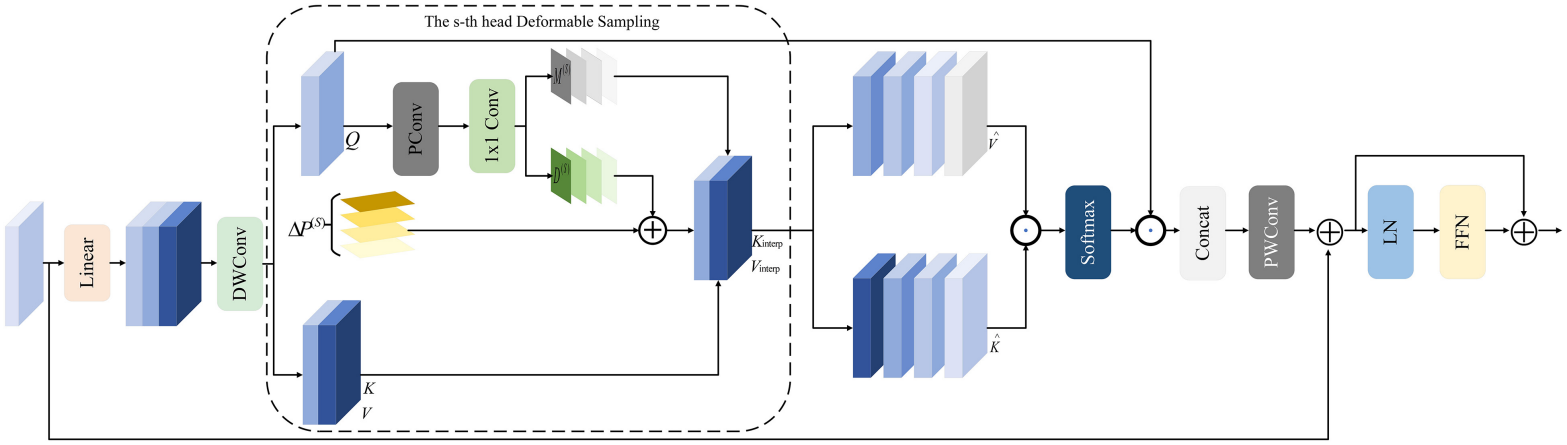
The MDSBlock architecture diagram.

Accurate geometric modeling of non-rigid cattle targets is a long-standing challenge in livestock vision tasks, with existing studies exploring 3D parametric models and graph convolutional networks for cattle shape modeling (32). Positioned in all skip connections between the encoder and decoder, the Multi-scale Deformable Sampling Block (MDSBlock) is designed to refine target boundary delineation through scale-adaptive spatial sampling and geometric deformation modeling. As illustrated in Figure 3.

Formally, let the input feature tensor from the encoder be *X* ∈ ℝ^*B* × *H* × *W* × *C*^, where *B* denotes the batch size, *H* and *W* represent the spatial height and width of the feature map, and *C* is the number of feature channels. The input feature is first transformed via a linear projection layer to align the channel dimension, followed by a depth-wise convolution (DWConv) to refine local spatial correlations and encode channel-wise spatial context. This process generates the query (*Q*), key (*K*), and value (*V*) representations for subsequent attention:


$$\begin{array}{l} Q=\text{DWCon}{\text{v}}_{Q}\left(\text{Linea}{\text{r}}_{Q}\left(X\right)\right) \end{array}$$ (5)

$$\begin{array}{l} K=\text{DWCon}{\text{v}}_{K}\left(\text{Linea}{\text{r}}_{K}\left(X\right)\right) \end{array}$$ (6)

$$\begin{array}{l} V=\text{DWCon}{\text{v}}_{V}\left(\text{Linea}{\text{r}}_{V}\left(X\right)\right) \end{array}$$ (7)

where *Q, K, V* ∈ ℝ^*B* × *H* × *W* × *C*^ denote the projected feature representations. These features are then split into *S* parallel attention heads along the channel dimension, with each head assigned a channel dimension of *C*_*h*_ = *C*/*S* (ensuring *C* is divisible by *S*). In our implementation, we set the number of heads *S* = 4 consistent with the multibranch grouped convolution design for multi-scale receptive field coverage.

To break through the limitation of fixed regular sampling grids in traditional attention mechanisms and realize adaptive modeling of target deformations across different scales, we design a multi-scale head-exclusive initial sampling grid and a global query-driven deformable offset prediction mechanism for each attention head. For the *s*-th attention head (*s* ∈ {1, 2, …, *S*}), we first define a multi-dilation initial sampling grid Δ*P*^(*s*)^ with head-specific dilation parameters. The grid is defined as:


$$\begin{array}{l} \text{Δ}P^{\left(s\right)}={\left\{-E_{s},0,+E_{s}\right\}}^{2} \end{array}$$ (8)

where *E*_*s*_ denotes the dilation coefficient of the *s*-th attention head. We set *E*_*s*_ = {1, 3, 5, 7} for the 4 parallel heads, enabling each head to capture geometric features at distinct receptive fields. The aggregation of multi-scale grids across heads forms a broad and sparse receptive field, which effectively captures long-range structural information of cattle targets while retaining fine-grained boundary details.

On this basis, we predict the deformable sampling offset field for each head under the guidance of the global query feature, rather than the split single-head query feature, to ensure the consistency of deformation modeling across heads and enhance the adaptability to non-uniform spatial distribution of target contours. For the *s*-th head, the spatial offset field *D*^(*s*)^ ∈ ℝ^*B* × 2*k*^^2^ × *H* × *W* and the deformation weight matrix *M*^(*s*)^ ∈ ℝ^*B* × *k*^^2^ × *H* × *W* are predicted in parallel, where *k* = 3 denotes the kernel size of the sampling window. The prediction process is defined as:


$$\begin{array}{l} D^{\left(s\right)}=\text{Con}{\text{v}}_{1\times 1}\left(\text{PCov}\left(Q\right)\right) \end{array}$$ (9)

$$\begin{array}{l} M^{\left(s\right)}=\text{Sigmoid}\left(\text{Con}{\text{v}}_{1\times 1}\left(\text{PCov}\left(Q\right)\right)\right) \end{array}$$ (10)

where PCov denotes partial convolution for local feature encoding, and the factor 2*k*^2^ in the offset dimension indicates that two offsets (Δ*x*, Δ*y*) are predicted for each of the *k*^2^ sampling positions within the window. The weight matrix *M*^(*s*)^ is constrained to the range [0, 1] via the sigmoid activation, serving as a learnable attention weight to filter invalid background sampling points and enhance the focus on discriminative regions such as cattle contours and edges.

With the multi-scale initial grid and deformable offset field, we generate the final adaptive sampling coordinates for the *s*-th head, which modulates the regular grid sampling positions to fit the geometric deformation of the target. The final sampling coordinate set at the central anchor of the sampling window *u* is defined as:


$$\begin{array}{l} P^{\left(s\right)}\left(u\right)=u+\text{Δ}P^{\left(s\right)}+D^{\left(s\right)} \end{array}$$ (11)

where *u* is the central spatial coordinate of the query, Δ*P*^(*s*)^ is the head-specific multi-scale initial sampling grid, and *D*^(*s*)^ is the deformable offset at position *u* for the *s*-th head. This formulation realizes the adaptive adjustment of sampling positions, enabling the module to dynamically align the sampling grid with the actual contour of cattle targets, even in the case of occlusion and pose deformation.

Subsequently, bilinear interpolation is performed on the raw *K* and *V* features at the deformable coordinates *P*^(*s*)^ weighted by the deformation weight matrix *M*^(*s*)^, to generate the calibrated key ${\hat{K}}_{s}$ and value ${\hat{V}}_{s}$ features. This adaptive sampling strategy enables the module to focus on discriminative contour regions rather than redundant background areas, facilitating precise capture of irregular deformation features of cattle. Scaled dot-product attention is then computed within each head for contextual feature aggregation:


$$\begin{array}{l} \text{Att}{\text{n}}_{s}=\text{Softmax}\left(\frac{Q_{s}\cdot {\hat{K}}_{s}^{⊤}}{\sqrt{C_{h}}}\right)\cdot {\hat{V}}_{s} \end{array}$$ (12)

where $\sqrt{C_{h}}$ serves as the scaling factor to mitigate gradient instability caused by excessive inner product values, and *Q*_*s*_ denotes the split query feature of the *s*-th head.

Outputs from all attention heads are concatenated along the channel dimension and fused via a point-wise convolution (PWConv). A residual connection is subsequently incorporated, followed by layer normalization (LN) and a feed-forward network (FFN), to generate the final output feature.

### 2.4 The loss function

The loss function of the proposed MFDSU-Net consists of two terms, including the Dice loss (L_Dice_) and the Tversky loss (L_Tversky_), respectively. The total loss function is defined as:


$$\begin{array}{l} L=0.5\cdot L_{\text{Dice}}+0.5\cdot L_{\text{Tversky}} \end{array}$$ (13)

where L_Dice_ and L_Tversky_ are defined as follows:


$$\begin{array}{l} L_{Dice}=1-\frac{2\sum_{i}{ŷ}_{i}y_{i}+\epsilon }{\sum_{i}{ŷ}_{i}+\sum_{i}y_{i}+\epsilon } \end{array}$$ (14)

$$\begin{array}{l} L_{Tversky}=1-\frac{2\sum_{i}{ŷ}_{i}y_{i}+\epsilon }{\sum_{i}{ŷ}_{i}y_{i}+\alpha \sum_{i}\left(1-{ŷ}_{i}\right)y_{i}+\left(1-\alpha \right)\sum_{i}{ŷ}_{i}\left(1-y_{i}\right)+\epsilon } \end{array}$$ (15)

where ŷ_*i*_ ∈ [0, 1] denotes the predicted probability at pixel *i*, *y*_*i*_ ∈ {0, 1} is the corresponding ground truth label. In this study, ϵ represents a small constant that is utilized to prevent division by zero and guarantee numerical stability during model training, which is empirically set to 10^−15^. The hyperparameter α of the Tversky loss is empirically set to 0.7.

Specifically, Dice loss is suited for segmentation tasks with imbalanced foreground-background pixel distributions, as it directly optimizes region overlap rather than pixel-wise classification. In contrast, Tversky loss introduces a tunable weighting mechanism to balance false positives and false negatives, which enables more precise control over boundary-sensitive segmentation performance.

### 2.5 Evaluation metrics

To quantitatively evaluate the segmentation performance of the proposed MFDSU-Net, two standard evaluation metrics were employed: Dice Similarity Coefficient (Dice) and Intersection over Union (IoU).

Specifically, Dice measures the overlap between predicted and ground-truth regions, and is defined as:


$$\begin{array}{l} Dice=\frac{2|P\cap G|}{\left|P|+|G\right|} \end{array}$$ (16)

where *P* denotes the predicted binary segmentation mask and *G* represents the ground-truth mask. |*P*∩*G*| is the number of overlapping pixels between the two regions, while |*P*|+|*G*| indicates the total number of pixels in both regions. The Dice value ranges from 0 (no overlap) to 1 (perfect overlap), with higher values indicating better segmentation accuracy.

In addition, IoU quantifies the ratio of intersection to union between predicted and ground-truth regions, and is formulated as:


$$\begin{array}{l} IoU=\frac{\left|P\cap G\right|}{\left|P\cup G\right|} \end{array}$$ (17)

where |*P*∩*G*| represents the number of pixels in the intersection of the predicted and ground-truth regions, and |*P*∪*G*| denotes the total number of pixels in their union. A higher IoU value reflects better agreement between the predicted segmentation and the ground truth.

## 3 Dataset and implementation details

### 3.1 Dataset

The experimental data were collected at the National Jinnan Cattle Genetic Resource Conservation Center in Yuncheng, Shanxi Province. All images were captured in standardized cattle barns and included Jinnan cattle at different growth stages. Jinnan cattle were selected in this study due to their representative large-scale breeding conditions and substantial posture variability under practical farm environments. In addition, the dataset contains diverse scenarios including illumination variation, inter-object occlusion, posture deformation, and complex background interference, which are highly representative of common challenges encountered in precision livestock farming. The core innovation of the proposed MFDSU-Net is the universal semantic-geometric collaborative architecture, which is not limited to a specific cattle breed. The multi-scale feature aggregation and adaptive deformable sampling modules are designed for general semantic enhancement and geometric deformation modeling, which can be extended to other cattle breeds and livestock species. To improve dataset diversity and reduce potential sample distribution bias, images were collected under different viewpoints, cattle behaviors, and spatial distributions within the breeding environment. To simulate the unconstrained imaging conditions typical of real-world breeding scenarios, a Canon EOS 1300D DSLR camera and a SEA-AL10 smartphone were used for multi-source data collection. All raw images had a resolution of at least 1, 920 × 1, 080 pixels.

A total of 8,433 raw images were obtained. After removing severely blurred images caused by focusing failures, 2,100 images exhibiting characteristics representative of real breeding environments were carefully selected to construct the experimental dataset. This dataset provides reliable data support for evaluating the cattle segmentation performance of the proposed MFDSU-Net model in complex pasture environments.

To guarantee the effectiveness of model training and the reliability of test results, stratified sampling was employed to divide the 2,100 annotated samples into training and test sets at an 8:2 ratio, resulting in 1,680 training samples and 420 test samples. Stratified sampling ensures consistent feature distribution between the training and test sets, thereby avoiding evaluation bias caused by uneven sample distribution. It enables the experimental results to truly reflect the cattle segmentation performance of MFDSU-Net in actual pasture scenes. To eliminate randomness arising from a single data split, a five-fold cross-validation strategy was adopted, and the average results over five runs are reported. All comparative models were trained and tested on the same five-fold partitions.

### 3.2 Training settings

The experiment was built based on the PyTorch deep learning framework. The hardware environment included an Intel Core i7-12700K CPU and an NVIDIA RTX 4090D 24G GPU, and the software environment included Ubuntu 18.04 operating system and CUDA 11.6.

All baseline models and the proposed MFDSU-Net were trained with a unified strategy to eliminate the influence of differences in training parameters on the experimental results. The AdamW optimizer was selected with an initial learning rate of 1 × 10^−4^, weight decay of 1 × 10^−5^, and a cosine annealing learning rate scheduling strategy was used to adjust the learning rate adaptively. The Dice loss and the Tversky loss were used to solve the class imbalance problem in the cattle segmentation task. The training batch size was set to 4, and the total training epoch was 200. Meanwhile, data augmentation methods such as random cropping, horizontal flipping, and adaptive adjustment of brightness and contrast were adopted to improve the generalization ability of the model.

Detailed hardware and software configurations are presented in Table 1.

**Table 1:** Configuration of hardware and software environment for experiments.

Item	Configuration
Operating system	Ubuntu 18.04
GPU	NVIDIA GeForce RTX 4090D (24 GB)
GPU environment	CUDA 11.6
CPU	Intel Core i7-12700K
Deep learning framework	PyTorch
Epochs	200
learning rate	1 × 10^−4^
weight decay	1 × 10^−5^
optimizer	AdamW

## 4 Experiments and analysis

### 4.1 Evaluate the effectiveness of different models

To fully verify the advantages and segmentation accuracy improvement of the proposed MFDSU-Net for cattle segmentation, we compared it against several representative models, including U-Net, Attention U-Net, U-Lite, TransU-Net, and MambaU-Lite. All models were trained and tested on the Jinnan cattle segmentation dataset under identical experimental settings and training strategies. All experimental protocols follow the state-of-the-art evaluation standards for precision livestock farming vision tasks (33), ensuring the comparability and credibility of our results. The corresponding results are presented in Table 2.

**Table 2:** Performance comparison of cattle segmentation models.

Model	Params (M)	FLOPs (G)	FPS	Dice (%)	IoU (%)
AttentionU-Net	2.400	1.34	58.86	88.01 ± 0.27	81.10 ± 0.24
U-Lite	0.627	1.25	67.28	87.88 ± 0.22	80.90 ± 0.31
U-Net	1.600	3.82	79.66	87.85 ± 0.26	80.86 ± 0.23
MambaU-Lite	0.416	0.93	44.52	87.64 ± 0.24	80.58 ± 0.33
TransU-Net	13.600	3.12	64.97	87.60 ± 0.31	80.44 ± 0.27
Ours (MFDSU-Net)	0.712	2.80	62.58	88.14 ± 0.17	81.38 ± 0.25

The quantitative comparison results are reported in Table 2. It can be observed that MFDSU-Net achieves competitive segmentation performance among lightweight models with fewer than 1M parameters. Specifically, MFDSU-Net achieves a Dice score of 88.14% and an IoU of 81.38%, which are 0.5 percentage points (pp) and 0.8 pp higher than those of MambaU-Lite, respectively. Statistical significance analysis further demonstrates that these improvements are not caused by stochastic training variations, with statistically significant differences observed for both Dice (*p* < 0.05) and IoU (*p* < 0.05). This indicates that the proposed model effectively improves segmentation accuracy under strict parameter constraints. Compared with conventional models such as U-Net and Attention U-Net, although they obtain competitive segmentation results, their parameter scales are significantly larger, leading to increased computational cost and reduced efficiency in practical deployment.

**Figure 4:**
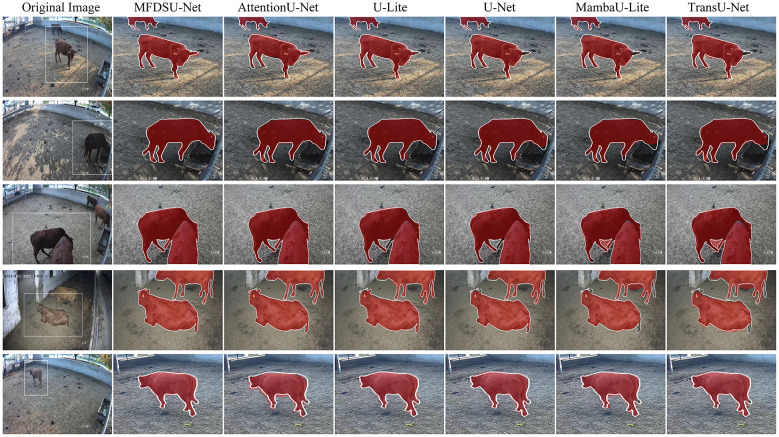
Visual comparison of cattle segmentation results obtained by various models in complex pasture scenes.

To provide an intuitive evaluation of segmentation performance, Figure 4 presents a visual comparison of different models on representative cattle images under complex farm scenarios. In each sample, the predicted segmentation regions are highlighted in red, while the ground-truth annotations are indicated in white. The spatial consistency between the red and white regions directly reflects the segmentation accuracy.

From the zoomed-in views, it can be observed that conventional models tend to suffer from boundary blurring and structural inconsistency, particularly in regions with ambiguous semantic cues or geometric deformation. In contrast, the proposed MFDSU-Net achieves closer alignment with the ground truth, exhibiting more coherent object structures and sharper boundary transitions. This improvement can be attributed to the collaborative design of MFABlock and MDSBlock, where MFABlock enhances semantic discrimination through multi-scale context aggregation, and MDSBlock refines geometric representation via adaptive deformable sampling. As a result, the model effectively mitigates semantic ambiguity while preserving fine-grained contour details, leading to superior visual segmentation quality.

Overall, the performance improvement can be attributed to the collaborative design of MFABlock and MDSBlock, which jointly enhance the semantic and geometric representation capabilities of the network. Specifically, MFABlock enhances semantic representation by aggregating multi-scale contextual information, enabling the model to better capture global context and alleviate foreground-background ambiguity in complex environments. Meanwhile, MDSBlock improves geometric modeling by adaptively adjusting sampling locations to object deformation, thereby enhancing boundary delineation and preserving fine-grained structural details. Through the complementary interaction of these two modules, MFDSU-Net effectively improves feature representation quality and achieves a favorable trade-off between segmentation accuracy and computational efficiency.

### 4.2 Evaluate the effectiveness of the internal core branch components in the MFABlock

To assess the contribution of the MFABlock module to cattle segmentation performance within MFDSU-Net, comparative experiments were conducted under eight distinct branch configurations. These configurations comprised the baseline setting with no MFABlock branches, the original-branch only setting, the 3 × 3 depthwise convolution branch only setting, the 5 × 5 depthwise convolution branch only setting, a dual-branch setting composed of the original and 3 × 3 depthwise convolution branches, a dual-branch setting composed of the original and 5 × 5 depthwise convolution branches, a dual-branch setting composed of the 5 × 5 depthwise convolution branches and 3 × 3 depthwise convolution branches, and the complete three-branch setting. The quantitative outcomes corresponding to each configuration are summarized in Table 3.

**Table 3:** Ablation study results for branches in MFABlock.

Original branch	3 × 3 branch	5 × 5 branch	Dice (%)	IoU (%)
×	×	×	87.64 ± 0.24	80.58 ± 0.33
✓	×	×	87.75 ± 0.21	80.69 ± 0.27
×	✓	×	87.72 ± 0.28	80.66 ± 0.22
×	×	✓	87.74 ± 0.19	80.68 ± 0.31
✓	✓	×	87.78 ± 0.23	80.71 ± 0.24
✓	×	✓	87.79 ± 0.17	80.72 ± 0.29
×	✓	✓	87.67 ± 0.30	80.61 ± 0.26
✓	✓	✓	87.84 ± 0.16	80.83 ± 0.23

The results presented in Table 3 demonstrate that varying the branch configuration yields distinct segmentation outcomes. Relative to the baseline, each single-branch variant of the MFABlock yields improvements in both the Dice coefficient and Intersection over Union. Among the single-branch configurations, the setting employing solely the original branch attains the highest individual performance. When the original branch is paired with a single depthwise convolution branch, the model achieves further performance gains. Specifically, the dual-branch configuration incorporating the original branch and the 5 × 5 depthwise convolution branch outperforms the configuration utilizing the original branch and the 3 × 3 depthwise convolution branch. The most favorable results are obtained upon the concurrent integration of all three branches. Under this full three-branch configuration, the Dice coefficient reaches 87.84% and the Intersection over Union attains 80.83%. These values correspond to absolute increases of 0.20 percentage points in Dice and 0.25 percentage points in IoU over the baseline.

Overall, the advantage conferred by the MFABlock resides in the collaborative representation of global semantic information and multi-scale local features. The original branch, which employs linear attention modeling, preserves global semantic coherence and captures long-range spatial dependencies inherent to cattle targets. Concurrently, the 3 × 3 depthwise convolution branch enhances the model's sensitivity to local edge textures and fine boundary details through depthwise separable convolution operations. The 5 × 5 depthwise convolution branch, by virtue of its enlarged receptive field, strengthens the model's capacity to accommodate target scale variations and to mitigate interference arising from complex farm background elements. The joint integration of these three branches enriches the representation of both the overarching semantic structure and the fine-grained local details of cattle instances, thereby elevating the Dice and IoU metrics. These results indicate that the MFABlock improves cattle segmentation performance in challenging farm environments through the concurrent enhancement of global semantic discrimination and multi-scale local feature representation. This mechanism effectively alleviates the semantic ambiguity encountered in the segmentation task.

### 4.3 Evaluate the effectiveness of the internal core functional components in the MDSBlock

To assess the contribution of the Multi-scale Deformable Sampling Block (MDSBlock) to cattle segmentation in MFDSU-Net, comparative experiments were conducted under four configurations: the Multi-scale Dilated Sampling Grid (MDSG) setting, the Query-guided Deformable Offset (QDO) setting, the integrated setting of both components, and the baseline setting where both components are removed. The corresponding results are presented in Table 4.

**Table 4:** Ablation study results for core components in MDSBlock.

MDSG	QDO	Dice (%)	IoU (%)
×	×	87.64 ± 0.24	80.58 ± 0.33
✓	×	87.86 ± 0.18	80.87 ± 0.25
×	✓	87.84 ± 0.22	80.83 ± 0.28
✓	✓	87.92 ± 0.19	81.04 ± 0.24

The results in Table 4 demonstrate that different component configurations yield distinct geometric modeling performance. The Multi-scale Dilated Sampling Grid (MDSG) alone achieves better performance than the Query-guided Deformable Offset (QDO) alone, and their combination delivers the optimal segmentation effect.

The superior performance of MDSG over QDO can be attributed to its explicit multi-scale spatial prior: the head-specific dilated sampling grids provide a broad and structured receptive field coverage, which inherently preserves the global shape integrity of cattle targets even under partial occlusion. In contrast, QDO relies solely on data-driven offset prediction without explicit spatial constraints, leading to unstable sampling when dealing with severely deformed or occluded regions.

When integrated, the two components exhibit strong complementary effects: MDSG provides a robust global geometric foundation, while QDO dynamically adjusts local sampling positions to capture fine-grained contour details. This collaborative mechanism enables MDSBlock to simultaneously maintain large-scale shape consistency and small-scale boundary precision, which is critical for accurate segmentation of non-rigid cattle targets.

Overall, the advantage of MDSBlock derives from the collaborative modeling of multi-scale receptive fields and adaptive deformation sampling. The Multi-scale Dilated Sampling Grid through multi-scale dilated sampling grids rather than standard convolutional operations, maintaining spatial consistency across different object sizes. Meanwhile, the Query-guided Deformable Offset enables spatially adaptive sampling by predicting offsets conditioned on global query features and shape deformation through learnable offset prediction. When the two modules are introduced together, they enhance the representation of both global shape integrity and fine-grained boundary details, thereby improving Dice and IoU. These results indicate that MDSBlock improves segmentation performance by jointly preserving multi-scale structural information coupled with strengthening local geometric adaptability.

### 4.4 Ablation experiments

To verify the contributions of the core modules MFABlock and MDSBlock, as well as their combined effect on the cattle segmentation performance of MFDSU-Net, a series of ablation experiments were designed using the lightweight MambaU-Lite as the baseline model. In these experiments, MFABlock and MDSBlock were added to the baseline individually and then in combination, and finally the two modules were fused. All ablation models were trained and validated under the same experimental environment, training strategy and test set.

**Figure 5:**
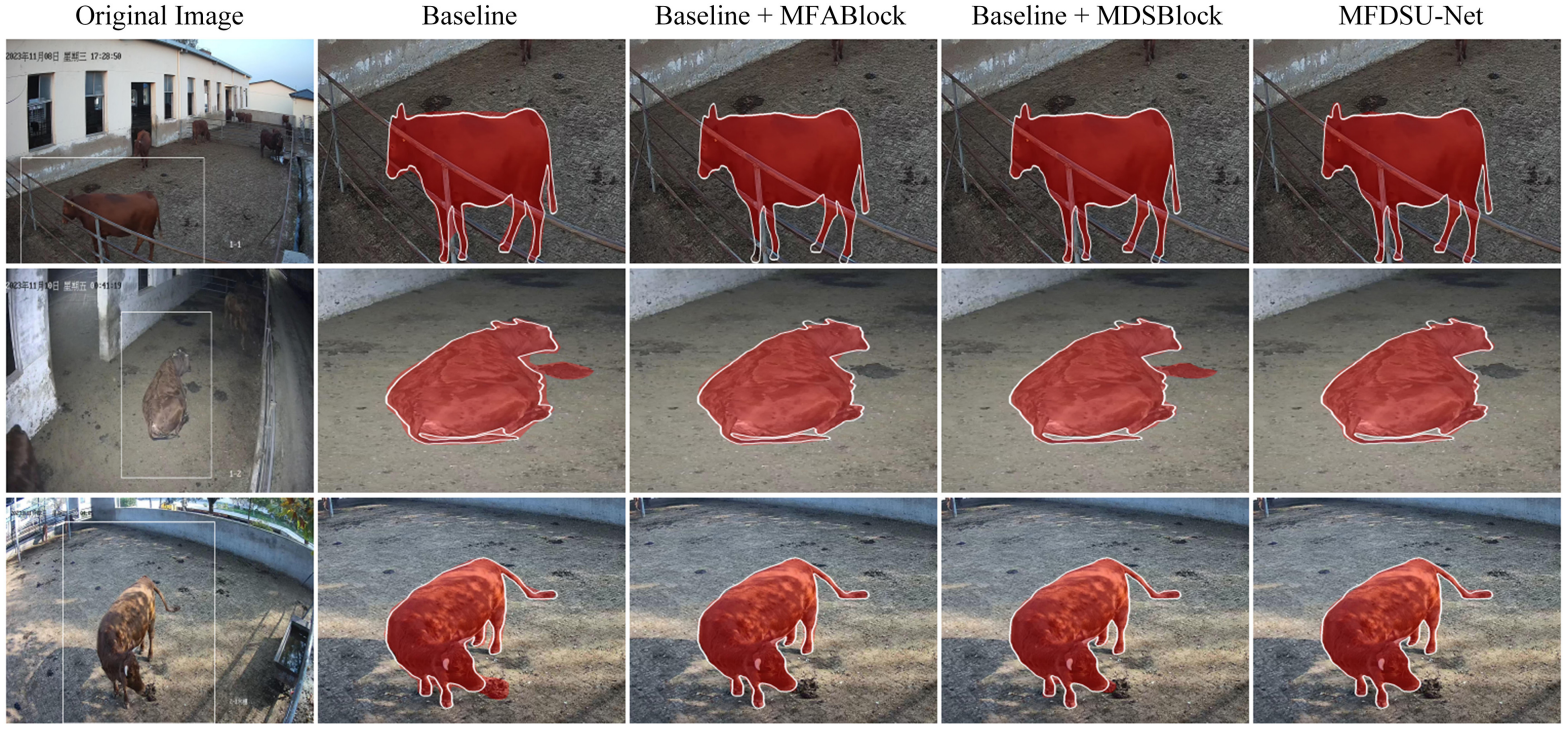
Qualitative comparison results of the ablation experiment.

The ablation results are shown in Table 5. When MFABlock is introduced into the baseline, the Dice score increases by 0.2% (*p* < 0.05), indicating that multi-scale feature aggregation effectively enhances semantic representation. When MDSBlock is incorporated, the Dice and IoU scores improve by 0.28% (*p* < 0.05) and 0.46% (*p* < 0.05), respectively. This demonstrates that adaptive deformable sampling contributes to better geometric modeling and more accurate boundary delineation. When both MFABlock and MDSBlock are integrated, the model achieves further improvements of 0.5% in Dice (*p* < 0.05) and 0.8% in IoU (*p* < 0.05), exceeding the gains obtained by each individual component. This result suggests that semantic enhancement and geometric modeling are complementary, and their combination leads to more robust segmentation performance under complex conditions.To intuitively verify the contribution of each core module to the segmentation performance, we present the qualitative comparison results of the ablation experiment in Figure 5.

**Table 5:** Ablation study results for MFDSU-Net.

Model	MFABlock	MDSBlock	Params (M)	Dice (%)	IoU (%)
Baseline	×	×	0.416	87.64 ± 0.24	80.58 ± 0.33
Baseline + MFABlock (Ours)	✓	×	0.477	87.84 ± 0.16	80.83 ± 0.23
Baseline + MDSBlock (Ours)	×	✓	0.651	87.92 ± 0.19	81.04 ± 0.24
MFDSU-Net (Ours)	✓	✓	0.712	88.14 ± 0.17	81.38 ± 0.25

## 5 Discussion

### 5.1 Semantic-geometric collaborative mechanism

This study proposes MFDSU-Net, a novel semantic-geometric segmentation framework designed for cattle instance extraction under complex farm environments. Trained on a dataset of 2,100 pixel-level annotated images, the proposed model achieves a favorable trade-off between segmentation accuracy and computational efficiency. Such a balance is particularly important for deployment on resource-constrained edge devices in precision livestock farming. Experimental results demonstrate that MFDSU-Net maintains strong robustness under challenging conditions, such as cluttered environments, articulated shape variations, and occlusion-induced information loss. Compared with existing lightweight segmentation approaches, as well as representative CNN-based, Transformer-based, and SSM-based models, the proposed method achieves superior segmentation performance while maintaining a significantly smaller parameter scale.

The effectiveness of MFDSU-Net stems from the collaborative design of its two core modules, which jointly enhance semantic representation and geometric modeling. The Multi-Scale Feature Aggregation Block (MFABlock) plays a key role in improving semantic discrimination by integrating contextual information across multiple receptive fields. This design enables the network to better distinguish cattle regions from complex and cluttered backgrounds, where traditional convolutional operations often struggle due to limited contextual awareness. By aggregating multi-scale features, MFABlock enhances robustness to scale variation and reduces semantic ambiguity caused by background noise.

Meanwhile, the Multi-scale Deformation Sampling Block (MDSBlock) focuses on modeling geometric variations and spatial deformations. In real-world farm conditions, cattle frequently exhibit non-rigid motion, diverse poses, and partial occlusion, which significantly complicate accurate boundary localization. Conventional fixed-grid convolution is inherently limited in capturing such irregular structures. To address this issue, MDSBlock introduces multi-scale deformable sampling, allowing the network to adaptively adjust sampling locations according to object geometry. This mechanism facilitates more precise feature aggregation under varying shapes and occlusion patterns. As a result, the model achieves improved boundary continuity and preserves both fine-grained contour details and global structural consistency.

The complementary interaction between MFABlock and MDSBlock highlights the importance of jointly considering semantic and geometric information in segmentation tasks. While MFABlock strengthens feature representation in the semantic domain, MDSBlock enhances spatial adaptability and structural coherence. The observed performance gains indicate that neither semantic enhancement nor geometric modeling alone is sufficient; instead, their coordinated integration is essential for achieving accurate segmentation in complex agricultural scenes.

In comparison with conventional CNN-based architectures such as U-Net and its variants, MFDSU-Net demonstrates improved capability in maintaining complete object structures, particularly under occlusion and low-contrast conditions. Although CNN-based methods are effective at extracting local features, their limited receptive fields restrict their ability to model long-range dependencies. Transformer-based models, on the other hand, can capture global context more effectively but typically incur higher computational cost and parameter overhead. Relative to these approaches, MFDSU-Net achieves a more balanced compromise between accuracy and efficiency. Furthermore, compared with SSM-based architectures such as MambaU-Lite, the proposed model exhibits stronger preservation of local geometric details due to its explicit deformable sampling strategy, which is critical for precise contour delineation.

### 5.2 Limitations

Despite the promising performance achieved by MFDSU-Net, several limitations still remain. First, the current dataset is limited to a single cattle breed, and the cross-breed generalization capability of the proposed framework has not yet been fully validated. Second, segmentation accuracy still degrades under extremely challenging scenarios involving severe foreground-background similarity and heavy inter-object occlusion. In particular, performance degradation can be observed when the visible overlap between cattle targets exceeds approximately 50%. Third, the current experiments are mainly conducted in standardized indoor breeding environments, while outdoor grazing environments with varying illumination conditions, nighttime scenes, rain, and fog remain unexplored. Finally, this study focuses on static image segmentation and does not incorporate temporal information from video sequences, which could potentially improve segmentation stability and motion-aware representation.

Nevertheless, these limitations do not diminish the overall contribution of this work. The semantic-geometric collaborative design of MFDSU-Net offers a generalizable framework that has the potential to be extended to other livestock segmentation tasks. The cross-breed and cross-species generalization performance of the model will be systematically verified in our follow-up research with multi-breed and multi-species datasets.

In summary, this study validates the feasibility of constructing a novel segmentation network that simultaneously maintains high accuracy and efficiency in complex farm environments. MFDSU-Net provides an effective and practical solution for cattle segmentation and shows strong potential for integration into intelligent livestock monitoring systems.

## 6 Conclusion

This paper presents MFDSU-Net, a novel network for cattle segmentation enhancement, which leverages both semantic and geometric information. Specifically, the MFABlock is introduced to capture contextual semantic information across varying receptive fields, thereby strengthening foreground-background discrimination. Meanwhile, the MDSBlock employs multi-scale deformable sampling to model spatial deformations at multiple granularities, enhancing the consistency between large-scale global shapes and small-scale local details. This design enables the network to better capture both fine-grained contour details and global shape variations, producing more complete and geometrically coherent segmentation results. Therefore, the MFDSU-Net improves cattle segmentation performance in complex farm scenarios. The experimental results show that the proposed MFDSU-Net achieves a Dice of 88.14% and an IoU of 81.38%, exceeding the existing state-of-the-art model by 0.5% and 0.8%, respectively. Moreover, with only 0.7M parameters and 2.8 GFLOPs, MFDSU-Net achieves a favorable balance between segmentation accuracy and efficiency.

Future work will extend the dataset to encompass multiple cattle breeds and diverse farming environments, thereby assessing the generalization capacity of the proposed framework across varied agricultural settings. Further investigation will also explore the applicability of the semantic-geometric collaborative design to other livestock segmentation tasks, such as sheep and pig, to establish a more universal lightweight solution for intelligent animal farming.

## Data Availability

The raw data supporting the conclusions of this article will be made available by the authors, without undue reservation.
